# Polyploid giant cancer cells and cancer progression

**DOI:** 10.3389/fcell.2022.1017588

**Published:** 2022-10-05

**Authors:** Xinyue Zhou, Mingming Zhou, Minying Zheng, Shifeng Tian, Xiaohui Yang, Yidi Ning, Yuwei Li, Shiwu Zhang

**Affiliations:** ^1^ Graduate School, Tianjin Medical University, Tianjin, China; ^2^ Department of Pathology, Tianjin Union Medical Center, Tianjin, China; ^3^ Nankai University School of Medicine, Nankai University, Tianjin, China; ^4^ Department of Colorectal Surgery, Tianjin Union Medical Center, Tianjin, China

**Keywords:** polyploid giant cancer cells, cancer stem cells, epithelial-mesenchymal transition, chemoradiotherapy resistance, tumor budding, micropapillary pattern

## Abstract

Polyploid giant cancer cells **(**PGCCs) are an important feature of cellular atypia, the detailed mechanisms of their formation and function remain unclear. PGCCs were previously thought to be derived from repeated mitosis/cytokinesis failure, with no intrinsic ability to proliferate and divide. However, recently, PGCCs have been confirmed to have cancer stem cell (CSC)-like characteristics, and generate progeny cells through asymmetric division, which express epithelial-mesenchymal transition-related markers to promote invasion and migration. The formation of PGCCs can be attributed to multiple stimulating factors, including hypoxia, chemotherapeutic reagents, and radiation, can induce the formation of PGCCs, by regulating the cell cycle and cell fusion-related protein expression. The properties of CSCs suggest that PGCCs can be induced to differentiate into non-tumor cells, and produce erythrocytes composed of embryonic hemoglobin, which have a high affinity for oxygen, and thereby allow PGCCs survival from the severe hypoxia. The number of PGCCs is associated with metastasis, chemoradiotherapy resistance, and recurrence of malignant tumors. Targeting relevant proteins or signaling pathways related with the formation and transdifferentiation of adipose tissue and cartilage in PGCCs may provide new strategies for solid tumor therapy.

## Introduction

Large-sized cells distributed in malignant tumor tissues have received little attention. This type of cell is usually thought to be senescent. The main reason is that large-sized cells are considered to be derived from repeated mitosis/cell division failure or genomic instability intermediates, and cannot maintain long-term survival and proliferation (Geigl et al., 2008; Holland and Cleveland, 2009). Large cells have been described using a variety of terms, such as polyaneuploid cancer cells, osteoclast-like cancer cells, pleomorphic cancer cells, blastomere cancer cells, etc. Zhang, S. et al. used cobalt chloride (CoCl_2_) to successfully purify a single polyploid giant cancer cell (PGCC), and described the formation of PGCCs and their roles in the invasion, migration, and chemoresistance of malignant tumors (Zhang et al., 2014a).

PGCCs can be induced by many kinds of stresses just like hypoxia, radiation, chemical drugs, virus and other stimuli that lead to DNA double-strand breaks (Fei et al., 2019a; Song et al., 2021; Nehme et al., 2022). PGCCs have cancer stem cell (CSC)-like characteristics and can express the CSC-related markers (CD44 and CD133) (Zhang et al., 2014a). PGCCs can rapidly produce small-sized progeny cells through asymmetric divisions, including budding, splitting, and bursting (Zhang et al., 2014b). Moreover, PGCCs can also produce tumor stromal cells, including erythrocytes, fibroblasts, skeletal muscular cells, adipocytes, and endothelial cells (Ganem and Pellman, 2007; Zhang et al., 2013a; Zhang et al., 2014a; Niu et al., 2017). In terms of tumor metastasis and invasion, PGCCs-derived tumors express epithelial-mesenchymal transition (EMT)-related proteins, including N-cadherin, snail/slug, and twist, which enhance the ability of cancer to metastasize and invade (Wang et al., 2019). In addition, PGCCs can be related to chemoresistance, tumor recurrence, and poor prognosis of solid cancer (Jia et al., 2012; Zhang et al., 2014a; Zhang et al., 2015). PGCCs appear in many types of malignant solid tumors, including melanoma, urothelial cancer, kidney cancer, breast cancer, ovarian cancer, pancreatic cancer and prostate cancer (Nehme et al., 2022; Fei et al., 2015; Liu et al., 2018; Lv et al., 2014; Zhang et al., 2014c; Imai et al., 1999; Lorentzen, 1980; Molberg et al., 1998; Mosnier and Balique, 2000; Nai et al., 2005; O'Connor et al., 2002; Pokieser et al., 2003; Sakai et al., 2000; Lopez-Beltran et al., 2005; Shen and Wen, 2004; Amend et al., 2019; Nehme et al., 2021) (Table 1). The number of PGCCs in poorly differentiated was found to be greater than that in well-differentiated and poorly malignant tumors. Higher number of PGCCs has been observed in lymph node metastatic foci than that in primary tumor tissues. For the same cancer patient, the amount of PGCCs in the recurrent tumor tissue after treatment (radiotherapy and/or chemotherapy) was greater than that of pre-treatment (Liu et al., 2020a; Li et al., 2021; Zhao et al., 2021).

**TABLE 1:** PGCCs appeared in different types of cancer cell lines and human malignant tumors.

Cancer cell lines and human malignant tumors	Stresses
Ovarian cancer cell lines HEY, SKOv3	CoCl_2_, cisplatin (Zhang et al., 2014a; Zhang et al., 2014d)
Colorectal cancer cell lines LoVo, Hct-116	CoCl_2_, capecitabine, oxaliplatin, irinotecan, irradiation, bufalin (Fei et al., 2019a; Zhao et al., 2021)
Breast cancer cell lines MCF-7, MDA-MB-231, BT-549	CoCl_2_, paclitaxel, triptolide (Jia et al., 2012; Wang et al., 2019; Liu et al., 2020a; Liu et al., 2020b)
Human melanomas	PGCCs existed in primary anorectal malignant melanomas and their number was positively correlated with volume of tumor, and lymph node metastasis (Liu et al., 2018)
ovarian cancer	PGCCs were detected in human serous ovarian carcinomas and the number of PGCCs were positively correlate to tumor grade (Lorentzen, 1980; Zhang et al., 2014c; Lv et al., 2014)
pancreatic cancer	Osteoclast-like giant cells appeared in pancreatic carcinomas (Molberg et al., 1998; Imai et al., 1999; Nai et al., 2005)
esophageal cancer	PGCCs appeared in a case of esophageal pleomorphic (giant cell) carcinoma with neuroendocrine differentiation (Mosnier and Balique, 2000).
urothelial cancer	PGCCs were observed in recurrent giant cell tumor of the bladder (O'Connor et al., 2002).
renal cell carcinoma	Syncytial giant cells appeared in a case of renal cell carcinoma (Shen and Wen, 2004).
breast cancer	The number of PGCCs was correlated with EMT-related proteins in breast cancer (Fei et al., 2015).
	PGCCs emerged in mammary epithelial cells infected with high-risk human cytomegalovirus (Nehme et al., 2021; Nehme et al., 2022).
prostate cancer	Two cases of pleomorphic giant cell carcinoma of the prostate with poor prognosis were reported (Lopez-Beltran et al., 2005)

## Polyploid, aneuploidy, and chromosome instability

Polyploidy describes an increase in the overall DNA content of the cell genome, and refers to the integral duplication of one or more complete chromosomes, such as 4N, 5N, and 12N. Aneuploidy indicates an abnormal number of chromosomes or chromosome fragments (Ben-David and Amon, 2020). Moreover, poly-aneuploids indicate one or more integral increases in the whole aneuploid genome (>4N+). Polyploidy can result in shifts in genetic material to get a greater chance of generating new beneficial mutation. In addition, polyploidy can be accompanied by phenotypic changes that have adaptive potential and allow niche partitioning between newly formed species and their ancestors (Otto and Whitton, 2000). Cancer cells with a relatively high DNA content usually appear as 4N+, 6N+, or higher, and exist in the form of poly-aneuploidy. Cancer species may exist in the state of 2N+ and poly-aneuploidy, with a branched cancer cell capable of transferring between these two states. As poly-aneuploids recover to the 2N+ state, chromosomes or chromosome segments retained in odd numbers may be more suitable than those that are not (Pienta et al., 2020).

Chromosomal instability is one of the factors causing aneuploidy, which can promote tumorigenesis by increasing genetic heterogeneity. However, this form of aneuploidy is not a common promoter of tumorigenesis. In contrast, aneuploidy is a carcinogenic event that depends on the environment and type of cancer, and may be clinically relevant as a potential therapeutic target. Due to the general adaptive loss of aneuploidy, tumor aneuploidy may be the product of both positively and negatively selected forms, which are determined by the interactions among tumor stage, cell type, genomic background, microenvironment, and the immune system (Ben-David and Amon, 2020). The Cancer Genome Atlas and other genomic studies have clearly shown that human tumors are not merely a collection of mutated oncogenes and tumor suppressor genes. Almost all tumors show aneuploidy to some extent, and the frequency of copy number changes is comparable to the mutation frequency of key cancer genes (Taylor et al., 2018). Alterations in chromosome copy number contribute to large-scale phenotypic changes that enable cells to grow in different cellular environments, including metastatic sites (Vasudevan et al., 2021). In colon cancer, slow cell division and growth of primary xenografts can be caused by the acquisition of an additional copy of chromosome 5 (Sheltzer et al., 2017). However, aneuploidy can also lead to a partial EMT, thereby increasing cell invasion and metastasis. These phenotypes are unique and EMT cannot be observed when several other aneuploids are imported into the same cancer cell line. However, when the additional copy of chromosome 5 is introduced into other cancer cell lines, it fails to replicate its observed role in colon cancer (Vasudevan et al., 2020). Aneuploidy is involved in the transition of epithelial-mesenchymal phenotype. In ovarian cancer, the loss of chromosome arm 16p is associated with EMT, which promotes cell proliferation, whereas the loss of chromosome arm 10p can promote cell growth (Gao et al., 2016).

## Stimuli inducing formation of PGCCs

CoCl_2_ mimics hypoxia-induced cellular responses *in vitro* and activates hypoxia-mediated signaling pathways. PGCCs were induced upon treatment of many kinds of cancer cells with CoCl_2_ (Lopez-Sanchez et al., 2014). The expression level of the hypoxia-inducible factor (HIF)-1α subunit is low in cancer cells under normal oxygen saturation, because HIF-1α subunits are degraded by ubiquitin-protease hydrolysis complexes, immediately after translation. In a hypoxic microenvironment, HIF-1α degradation is inhibited, and HIF-1α, 1β subunits combine to form active HIF-1, which is then transferred into the nucleus, to regulate the transcription of multiple genes and promote cellular adaptation to hypoxia (Keith and Simon, 2007). CoCl_2_ stabilizes HIF-1α by inhibiting proline hydroxylase (Ho and Bunn, 1996). Paclitaxel is commonly used to treat various human malignancies (Bharadwaj and Yu, 2004). PGCC formation was induced by treatment of the human ovarian cancer cell line SKOv3 with 1 μM paclitaxel. PGCCs with daughter cells (PDCs) derived from paclitaxel treatment express the EMT phenotype, with increased expression of fibronectin, N-cadherin, vimentin, slug, and twist (Jia et al., 2012). Arsenic trioxide (ATO) can also induce the formation of PGCCs through regulating the expression of glial cell missing 1 (GCM1)-/syncytin1 in the colon cancer cell lines. Li et al. treated LoVo cells and HCT-116 cells with ATO. After treatment, most small-sized cancer cells were dead, and the surviving cells showed significant morphological changes (Li et al., 2021). Human cytomegalovirus can also induce the dedifferentiation of mature human mammary epithelial cells which possess PGCCs phenotype, and the number of PGCCs is positively correlates with EZH2/Myc expression in breast cancer (Nehme et al., 2021; Nehme et al., 2022). Overexpression of phosphatase of regenerating liver 3 (PRL3) can promote PGCC formation. Overexpression of PRL3 uncouples the cell cycle from DNA replication and induces PGCCs formation, which contributes to a significant increase in the ploidy of progeny cells rather than an increase in cell number. Moreover, overexpressed PRL3 inhibits the ataxia telangiectasia mutated (ATM)-related DNA damage, thereby allowing PGCCs to survive (Thura et al., 2021). ATM is a kind of kinases that activates cell cycle checkpoints after DNA damage. ATM kinase can phosphorylate various proteins that are involved in the regulation of cell cycle checkpoints, apoptosis, and DNA repair, such as checkpoint kinase 2 (CHK2), which is phosphorylated by ATM at the initiation site T68 (Zannini et al., 2014). Triptolide, which has anti-inflammatory and anticancer activities, is a compound extracted from Tripterygium. It induces the breast cancer lines BT-549 and HEY to form PGCCs (Wang et al., 2019). In addition, chemotherapy drugs, including capecitabine, oxaliplatin, and irinotecan, as well as radiation can also induce the formation of PGCCs. The presence of PGCC-rich tumor tissue is associated with recurrence, metastasis, chemoresistance, and poor prognosis (Fei et al., 2019a).

## Morphological characteristics of PGCCs

PGCCs constitute a unique subset of cancer cells. There are two types of PGCCs: multinucleated and mononucleated giant cancer cells. The common morphological characteristics of PGCCs are large nuclei (or multinuclei), and their average size is more than three times larger of diploid cancer cells (Zhang et al., 2014a). The nuclei of PGCCs are usually irregular, and the morphology of PGCCs varies among different cell lines. For example, PGCCs originating from HEY, MDA-MB-231, and HCT-116 cells have neuron-like morphologies. On the other hand, PGCCs derived from colon cancer Caco-2 are similar to PGCCs derived from ovarian SKOv3, with both being round, without cytoplasmic extension or branching (Lopez-Sanchez et al., 2014). The number of PGCCs can be used to predict prognosis and determine the degree of malignancy and differentiation of malignant solid tumors. In ovarian serous carcinoma, some researchers have proposed a two-level univariate system based on cell heteromorphism, to evaluate the grade.

## Endoreplication, cell fusion, and PGCC formation

**FIGURE 1:**
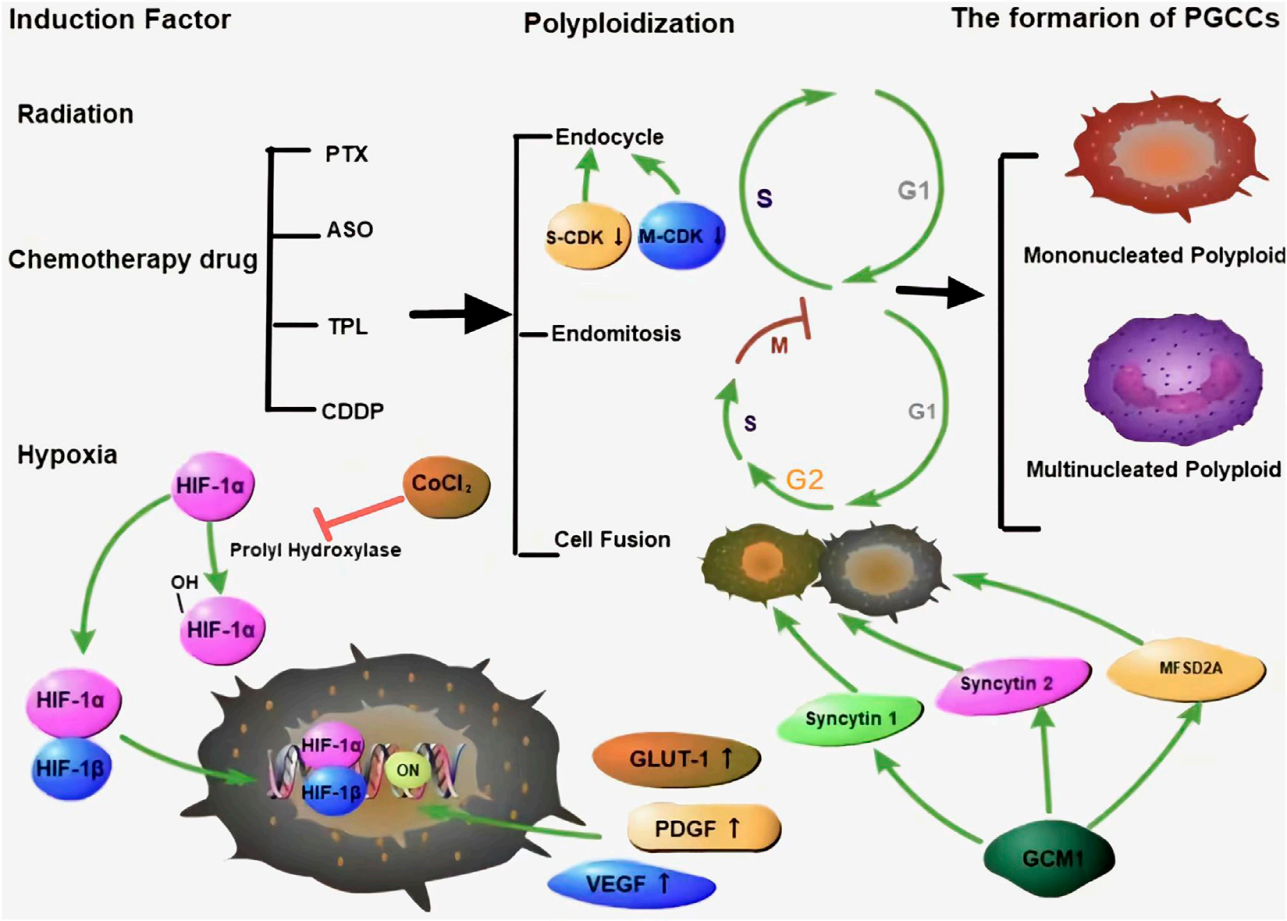
Many kinds of stresses can induce the formation of PGCCs, which can occur *via* endoreplication and cell fusion. There are two kinds of PGCCs, multinucleated and mononucleated giant cancer cells. HIF-1α is a key protein that is regulated by proline hydroxylase and is associated with the CSC-related characteristics of PGCCs. Green arrows represent potentiation, while red lines represent inhibitive effects.

Many chemical reagents and radiotherapy can induce PGCC formation through cell fusion or endoreplication. Under different stress conditions, polyploids can be induced in a variety of ways, with the degree of polyploidy varying even under the same stress. Both endoreduplication and cell fusion can induce the formation of PGCCs (Figure 1) (Niu et al., 2016; Liu, 2018; Øvrebø and Edgar, 2018; Chen et al., 2019; Moein et al., 2020).

There are two forms of endoreplication: endocycle formation and endomitosis. In *p53*/*Rb*-deletion cells, the G2 phase can be prolonged, and the cell cycle can be prevented from entering the M phase, by downregulating the levels of M-CDK, which is responsible for promoting G2/M phase progression and maintaining the S phase. With the periodic inactivation of S-CDK, the G and S phases are alternately driven in the pulse mode, which results in tumor cells entering the endocycle and forming polyploid cells (Harashima et al., 2013; Zielke et al., 2013; Edgar et al., 2014; Øvrebø and Edgar, 2018). Low levels of total CDK activation is important to maintain endocycle (Edgar et al., 2014). The endocycle is a small cycle that includes the G phase, S phase, and alternation between the two phases. The chromosomes and cytoplasm do not separate in the endocycle, which eventually results in the formation of mononuclear giant cells containing a large amount of DNA (Lee et al., 2009; Harashima et al., 2013; Edgar et al., 2014; Liu, 2018). In endomitosis, the cell cycle enters the mitotic stage and passes the G2/M checkpoint, with incomplete separation of sister chromatids and cytokinesis, which can also result in the formation of multinucleated giant cells (Lee et al., 2009).

Except for PGCC formation by abnormal mitosis, cell fusion can also associate with PGCCs (Song et al., 2021). Cell fusion plays a crucial role in muscle tissue formation, immune response, tissue repair, and is also involved in the progression of cancer, including origin of CSCs, drug resistance, and metastasis (Lu and Kang, 2009). Syncytin-1 and -2 are two critical proteins involved in cell fusion (Zhang et al., 2021). GCM1 is related to the expression of syncytin-1 and mediates the transformation of monocytic trophoblast cells into multinucleated syncytiotrophoblast cells (Yu et al., 2002; Liang et al., 2010). The fusion of BeWo cells was found to be inhibited upon silencing GCM1 (Baczyk et al., 2009). The mechanism by which GCM1 participates in the regulation of syncytin-1 expression may be associated with two GCM1-binding sites located in syncytin-1 gene 5′-long terminal repeat upstream. After binding, GCM1 activates the gene and increases the expression of the pre-syncytin-1 fusion protein, to promote cell fusion (Lin et al., 2005). Ectopic expression of GCM1 can also activate the expression of syncytin-2 and Major facilitator superfamily domain-containing 2A (Liang et al., 2010). Multiple signaling pathways including cAMP/PKA, mitogen-activated protein kinase (MAPK), Wnt, and c-Jun N-terminal kinase (JNK) regulated syncytin-1 expression during embryonic development (Knerr et al., 2005; Strick et al., 2007; Matsuura et al., 2011).

## Cell cycle-related proteins and PGCCs formation

DNA synthesis needs to reduce cyclin D1 to a low level during the S phase, following which the level of cyclin D1 increases when the cells enter into the G2 phase. Low expression of cyclin D1 in the G2 phase can result in cell cycle arrest. Some researchers have proposed cyclin D1 as a switch that regulates cell cycle progression (Stacey, 2003). The presence of mitogens can result in the activation of Ras in G2 phase. Activated Ras increases the expression of cyclin D1 in the G2 phase, and causes cells to enter the S phase in a Ras-independent manner, even when Ras activity is eliminated in the G1 phase (Hitomi and Stacey, 2001).

**FIGURE 2:**
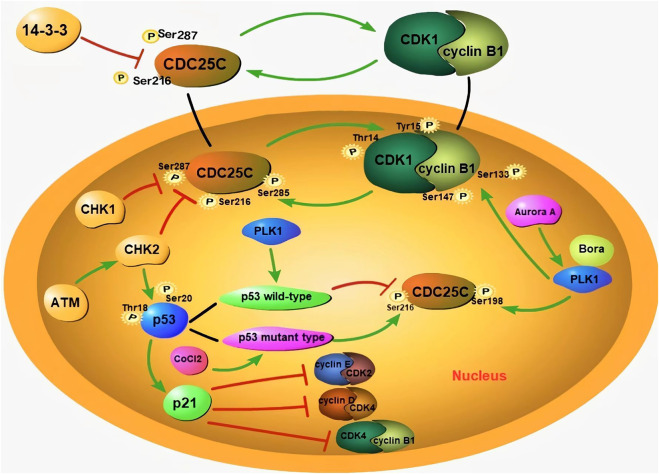
The formation of PGCCs is associated with aberrant expression subcellular localization of cell cycle-related proteins. During interphase, the phosphorylation of CDC25C at Ser216 and Ser287 sites can prevent the activation of CDC25C, by binding to 14-3-3 protein, to localize it in the cytoplasm. When CDC25C is activated, it can dephosphorylate CDK1 and activate nuclear entry of cyclin B1/CDK1. The phosphorylation site of CDC25C is associated with different *p53* genotypes. Wild-type p53 can inhibit the phosphorylation of CDC25C-Ser216, while mutant-type p53 can promote the same. The bind of p21 with cyclin E/CDK2 and cyclin D/CDK4 complexes can result in cell cycle G1 arrest. In addition, ATM activates CHK2, and results in the phosphorylation of CDC25C-Ser216. Phosphorylated CDC25C-Ser216 can combine with 14-3-3, to promote the cytoplasmic translocation of CDC25C. CHK2 is also able to phosphorylate P53, to promote the accumulation of p21 and maintain G2/M arrest. With the assistance of Bora, Aurora A phosphorylates PLK1, and promotes the nuclear localization of CDC25C. PLK1 can also phosphorylate the cyclin B1/CDK1 complex and promote its entry into the nucleus. Green arrows represent potentiation, while red lines represent inhibitive effects.

An abnormal cell cycle can lead to uncontrolled cell proliferation. Abnormal expression of cell cycle-related proteins enables tumor cells to have the abilities of invasion, metastasis, drug resistance, and anti-apoptosis. In recent years, several cell cycle-related proteins have emerged as potential biomarkers for the early diagnosis of malignancies and targets for cancer therapy. Several cell cycle-related proteins have been reported to be dysregulated during the formation of PGCCs. PGCC formation is associated with abnormal expression of cell cycle-related proteins including the expression level and sub-cellular localization. As shown in Figure 2, PGCCs derived post-CoCl_2_ treatment show low expression of CDC25C, cyclinB1, and CDK1 and high expression of PLK1, Aurora A, CHK2, and p53 (Liu et al., 2020b).

Abnormal expression of CDC25C is associated with tumorigenesis, metastasis, occurrence, and poor prognosis. The expression of CDC25C is regulated by cyclin B1/CDK1, PLK1, Aurora A, p53/Pin1, and ASK1/JNK/p38, etc (Liu et al., 2020b). The cytoplasmic localization of CDC25C depends on its amino acid sequence from residues 201–258 containing specific sites for binding to 14-3-3 proteins. The binding of 14-3-3 and CDC25C enables CDC25C to be localized in the cytoplasm (Sur and Agrawal, 2016). The phosphorylation of CDC25C at Ser216 and Ser287 sites can prevent the activation of CDC25C, by binding to the 14-3-3 protein, which results in its localization in the cytoplasm (Margolis et al., 2006). Boutros et al. demonstrated that the nuclear export sequence of CDC25C regulates its localization. The translocation of CDC25C can be promoted when Ser191 and Ser198 in the nuclear export sequence are phosphorylated (Boutros et al., 2007). During interphase, CDK1 is phosphorylated, which maintains the cyclinB1/CDK1 complex in an inactive state. CDC25C can dephosphorylate CDK1 by binding with the Thr14 and Tyr15 residues of CDK1, activate the nuclear entry of this complex, and promote cell mitosis (Sur and Agrawal, 2016). Phosphorylation at Ser285 is an important step in cyclin B1/CDK1-mediated positive feedback activation of CDC25C. Phosphorylation at this site blocks the CHK1-mediated phosphorylation of Ser287 and is beneficial for the maintenance of mitosis (Margolis et al., 2006).

P47 (P53 isoform lacking the first 39 amino acids) can regulate the arrest of the G2 phase cell cycle under different cellular stress conditions (Bourougaa et al., 2010). Treatment of colon cancer cells with CoCl_2_ induces the expression of p53/p47 and negatively regulates the progression of the G2 phase (Lopez-Sanchez et al., 2014). The tumor suppressor gene p53 can participate in G2/M phase cell cycle arrest by regulating CDC25C expression. P53 can monitor the G1 and G2/M phase check points and activate their transcriptional processes (Engeland, 2018). Two mechanisms are involved in the P53-mediated inhibition of CDC25C. First, the P53 represses the CDC25C promoter through CHR element, and p21 induces the downregulation of the CDC25C gene caused by DNA damage through this element. The other mechanism is related to the synergistic effect of transcriptional factor specificity protein 1 and P53 (Haugwitz et al., 2002; St Clair et al., 2004). The protein encoded by p21 is an inhibitor of CDK, results in cell cycle G1 arrest by binding to cyclin E-CDK2 and cyclin D-CDK4 complexes (Nosrati et al., 2015; Barr et al., 2017). At the same time, p21 can also promote the phosphorylation of the Tyr15 site of CDK1, to inhibit the activity of cyclinB1/CDK1 (Müllers et al., 2017). In addition, the phosphorylation of CDC25C is associated with the *p53* genotype. Kim et al. reported that wild-type p53 prevents polyploidy caused by mitotic failure by inhibiting the phosphorylation of CDC25C-Ser216 (Liu et al., 2020a). Mutant-type *p53* not only fails to exert its anticancer function, but also affects the normal function of wild-type p53 and promotes cancer progression (Baugh et al., 2018). Hypoxia can increase the expression of mutant p53, leading to G2/M arrest and PGCC formation (Liu et al., 2020a). Mutant-type *p53* can increase the phosphorylation of CDC25C-Ser216 and result in chromosome segregation failure (Kim et al., 2009).

ATM activates CHK2, which can phosphorylate the Ser216 and Ser287 residues of CDC25C. 14-3-3 can bind the two phosphorylated sites and promote CDC25C translocation to the cytoplasm. CHK2 is also able to phosphorylate Thr18 and Ser20 of P53, to promote the accumulation of p21 and maintain G2/M arrest (Liu et al., 2020b). Aurora A can phosphorylate PLK1 and promote CDC25C nuclear localization. Bora is a vital co-factor for Aurora A-mediated PLK1 activation by means of phosphorylation. The interaction between Bora and Plk1 leads to a conformational change that allows Aurora A to activate PLK1 (Liu et al., 2020b). PLK1 initiates the nuclear translocation of CDC25C by phosphorylating the Ser198 residue (Toyoshima-Morimoto et al., 2002). CDC25C is activated to dephosphorylate the cyclin B1/CDK1 complex. PLK1 can also phosphorylate the cyclin B1/CDK1 complex, to drive its translocation into the nucleus (Gheghiani et al., 2017).

## CSC-related characteristics of PGCCs

### Tumorigenicity of PGCCs

The sphere-forming experiment was used to measure the stemness of tumor cells. Zhang et al. (2014a) serially diluted PGCCs and then used three to five PGCCs on the Matrigel^®^. A single PGCC can form spheres on the 5^th^ day after culture, in Matrigel^®^. To determine the tumorigenicity of PGCC *in vivo*, A single PGCC was subcutaneously injected into nude mice, and each nude mouse was injected with a single spheroid, and all nude mice eventually developed xenografts. In addition, Zhang et al. (2014a) used NOD. CS17-Prkdc severe combined immunodeficiency mice to determine the ability of tumor formation and confirmed that a single PGCC could develop xenografts.

### Asymmetric cell division

In eukaryotes, mitosis is a well-established process of somatic cell division that ensures accurate segregation of replicated genetic material into progeny cells (Zhang et al., 2014b). ACD typically occurs in lower eukaryotes, plants, and viruses. The growth patterns of ACD in PGCCs include budding, splitting, and bursting. Daughter cell budding of PGCCs usually occurs in the branches of PGCCs and PGCCs containing multiple nuclei, followed by the sudden release of a large number of small PDCs (Zhang et al., 2014a). ACD is of great significance for cell diversity during normal tissue development (Horvitz and Herskowitz, 1992). In principle, two mechanisms including external and intrinsic ACD are involved in ACD. The external ACD means that the PDCs are equivalent at first, but are gradually differentiated by the surrounding cell-microenvironment-precursor cells. For intrinsic ACD, PDCs are essentially different at the time of mother cell division (Hawkins and Garriga, 1998). Intrinsic ACD rely on proteins, RNA transcripts, and macromolecules at different positions in progeny cells, leading to different fates of each cell and its brother cells (Zhang et al., 2014b). Cell cycle-related proteins and cell division control protein 42 (Cdc42) are associated with the generation of PDCs by regulating cytoskeletal changes and mitotic processes during cell division (Zhang et al., 2015).

The proteins involved in asymmetric division include FOXM1, cyclins, and Cdc42 (Zhang et al., 2014b). The peak expression of endogenous FOXM1 happens in the S and G2/M phases (Zhang et al., 2013b). The expression levels of cyclin E and cyclin D1 in PDCs are higher than those in the diploid cancer cells. Cyclin B1 expressing only located in the cytoplasm of PGCCs, indicating that PGCC formation is regulated by the redistribution of cyclins, which are usually involved in the regulation of ACD (Zhang et al., 2014a). Subcellular localization of cyclin B1 is associated with cell cycle progression. When cyclin B1 is phosphorylated, cyclin B1 is transferred to the nucleus (Müllers et al., 2014). The nuclear expression of cyclin B1 transits cells from G2 to M phase, whereas cytoplasmic expression of cyclin B1 can lead to G2/M phase arrest and PGCC formation (Fei et al., 2019b). As an important member of the Rho-GTP family, Cdc42 involves in multiple physiological processes by activating the JNK and P38/MAPK pathways. Zhang, S. et al. showed that Cdc42 is significantly upregulated in PGCCs and PDCs, as compared to that in control cells (Zhang et al., 2013b). In an unpublished study, we proved that Cdc42 is involved in the asymmetric division of PGCC-producing PDCs, by interacting with the microtubule polymeric protein, stathmin.

### Expression of CSC-related markers

CSCs are a subset of tumor cells with high plasticity, which are isolated using flow cytometry with CSC markers including CD24, CD44, CD133, ALDH, and Ep-CAM (Dianat-Moghadam et al., 2018). Tumor-spheroid formation *in vitro* is also associated with overexpression of CD44, CD133, Sox2, Nanog, and Oct-3/4 (Zhang et al., 2016). To determine whether PGCCs and PDCs can highly express CSC markers, Zhang, S. et al. confirmed that HEY and MDA-MB-231 PGCCs and PDCs highly express the CSC-related markers. Immunohistochemical staining confirmed the expression of CD44 and CD133 in PGCCs and their PDCs (Zhang et al., 2014a). Progenic cells derived from PGCCs express CSC-related markers and can be sorted using flow cytometry. In other words, the cells sorted using flow cytometry are a mixture of PGCCs and PDCs. The purification of PGCCs cannot be sorted using flow cytometry, and is possible following long-term treatment with high concentrations of CoCl_2_ (Zhang et al., 2014a).

### EMT-related proteins expression of PGCCs and PDCs

EMT can promote metastasis in human cancer (Mani et al., 2008). After EMT, the expression of epithelial cell markers decreases, while that of mesenchymal markers increases. Zhang, S. et al. proved that PGCCs and PDCs had low expression of epithelial phenotype markers, and high expression of mesenchymal phenotype markers in ovarian cancer (Zhang et al., 2014a). HIF-1α can induce twist expression to promote EMT and further promote tumor metastasis in PDCs, after CoCl_2_ treatment (Yang et al., 2008; Zhang et al., 2014a). In addition to expressing EMT-related proteins, the strong invasive and metastatic abilities of PDCs derived from PGCCs are associated with the sub-cellular location and post-translational modification of S100A10. S100A10 can bind to annexin A2, to form a heterotetrameric complex (AIIt) that is mainly localized in the cell membrane and cytoplasm. Phosphorylation of tyrosine 23 of S100A10 promotes the formation of AIIt (Zhao et al., 2021). AIIt is localized in the cytoplasm and can protect S100A10 from proteasomal degradation (He et al., 2008). AIIt can promote tissue plasminogen activator to cleave plasminogen to plasmin (Bresnick et al., 2015). S100A10 modified by SUMO can translocate into the nucleus and promote the proliferation, invasion, and metastatic abilities of PGCCs and PDCs, by regulating the expression of ARHGEF18, PTPRN2, and DEFA3 (Zhao et al., 2021). In addition, high expression levels of S100A4, cathepsin B, and cyclin B1 also play important roles in promoting the migration and invasion abilities of PGCC and PDCs. S100A4 is related to a variety of cytoskeletal proteins, and can promote tumor invasion and metastasis (Fei et al., 2017; He et al., 2017; Zhang et al., 2018; Fei et al., 2019a). S100A4-knockdown can inhibit the invasiveness of PDCs by decreasing the expression of cathepsin B and cyclinB1 (Fei et al., 2020).

### The ability of multilineage differentiation of PGCCs

Ishay-Ronen et al. reported that combinatorial treatment with mitogen-activated protein kinase inhibitors and the antidiabetic drug rosiglitazone induced the conversion of cancer cells expressing EMT-related proteins into post-mitotic adipocytes, thereby repressing the invasion and metastasis of primary tumor in breast cancer (Ishay-Ronen et al., 2019). EMT is known to enhance cellular plasticity, and can be exploited to enable transdifferentiation of cancer cells into functional adipocytes. The ability of cancer cells to transition between non-CSC and CSC states is called phenotypic plasticity (Das et al., 2020). The phenotypic plasticity of CSCs plays a vital role in the dynamic evolution of populations and tumor progression. PDCs derived from PGCCs possess EMT phenotypes and can differentiate into multilineage stromal cells (Zhang et al., 2014a).

Paclitaxel induces transformation of the ovarian cancer cells SKOv3 into a benign fibroblast phenotype, by regulating EMT-related proteins (Jia et al., 2012). Adipose and cartilage differentiation of PDCs was confirmed by culturing these cells in adipogenic and chondrogenic media, respectively (Zhang et al., 2014a). Upon subcutaneous injection of the animals with chondrogenic pellets, cartilage-like translucent nodules were observed inside the tumor mass. Histological examination and histochemical staining confirmed that PGCCs can differentiate into cartilage (Zhang et al., 2014a). In addition, MCF-7 PGCCs and PDCs formed spheroid-like structures after paclitaxel treatment. Histological examination showed that these spheroid-like structures had multicellular structures, including papillary, adenoid, and vascular morphologies. Immunohistochemical staining demonstrated that MCF-7 PDCs could differentiate into endothelial and myoepithelial cells (Zhang et al., 2014d).

### PGCCs can generate erythrocytes and form vasculogenic mimicry

PGCCs can generate erythrocytes upon CoCl_2_ treatment (Zhang et al., 2013a). Erythrocytes produced by PGCCs are composed of fetal and embryonic hemoglobin, and are different from the red blood cells produced by the bone marrow, which are composed of mature hemoglobin. Embryonic hemoglobin has a higher affinity for oxygen than mature hemoglobin, and thereby allow tumor cells to survive under severe hypoxic conditions (Kazazian and Woodhead, 1973; Huehns and Faroqui, 1975; Peschle et al., 1985; Albitar et al., 1992). VM is a pattern of blood supply to malignant tumors, wherein tumor cells constitute the structure of the vessel wall. Compared to that in well-differentiated tumors, more VMs appear in low-differentiated malignant tumors (Seftor et al., 2001; Seftor et al., 2002). Hypoxia can activate invasion and metastasis-related genes, which make the cells more aggressive, and induce them to form VM (Olenyuk et al., 2004; Shams et al., 2004). PGCCs and their generated fetal erythrocytes with high oxygen-binding affinity can form VM structures (Zhang et al., 2013a; Yang et al., 2018). Tumor cells lining the inner surface of the VM channel are directly exposed to the blood stream, which promotes hematogenous metastasis of tumor cells (Zhang et al., 2007).

### Slow cycle nature

The growth and division of PGCCs have unique features called the giant cell cycle including four different phases: initiation, self-renewal, termination, and stabilization (Niu et al., 2016). At the initiation phase, unsuccessful mitosis or cytokinesis can trigger endoreplication, enabling cells to evade senescence or apoptosis. At the stage of self-renewal, polyploid cells grow independently and evolve into morula-type, compacted type, and blastocyst-type embryos, giving rise to inner stem cells resembling endocytic clusters. At the termination stage, polyploid cells growth is completed, and the internal cell cluster-like stem cells generate small embryonic-like cells. At the stabilization stage, stem cells gradually acquire mitotic ability, stable diploid karyotypes, and differentiate into different grades of tumors (Niu et al., 2017). The degree of dedifferentiation alters in accordance with the varieties and duration of the stimulus, as well as cell type. As the giant cell cycle extends gradually, the resulting cells are closer to the initial cells, and have the greater potential to develop into tumors of different grades and types (Niu et al., 2017).

Zhang et al. (2014a) also explore the slow-cycle nature of PGCCs. *In vivo*, PGCCs labeled with PKH26 were subcutaneously injected into mice. Fluorescence was detected in the PGCCs, while there was no fluorescence in the PDCs, which was confirmed in the frozen slides of animal xenografts, indicating that the division of PGCCs was slow (Zhang et al., 2014a). Additionally, ELF-2pha was significantly downregulated in HEY PGCCs and SKOv3 PGCCs, as compared to that in cells that were not treated with CoCl_2_, suggesting that the protein synthesis rate of PGCCs was low (Zhang et al., 2013b).

### Chemoradiotherapy resistance of PGCCs and PDCs

Tumor heterogeneity is associated with chemoradiotherapy resistance. Highly heterogeneous tumors often contain a small number of clonal subsets that carry mutated genes in drug targets or proteins involved in apoptosis, senescence, or DNA repair. This mutation allows malignant cells to survive and repopulate tumors in response to chemoradiotherapy (Ogden et al., 2015). Some researchers have proposed that tumor cells with significantly increased genomic content can promote the rapid evolution of tumors and acquire therapeutic resistance during the treatment of various cancers. PGCCs are the main feature of tumor heterogeneity, and the number of PGCCs in recurrent tumor tissue after chemoradiotherapy is greater than that before treatment. PGCCs can evade cytotoxicity by entering a static state. After the completion of chemoradiotherapy, PGCCs can re-enter the cell cycle and produce PDCs with a chemoresistant phenotype. All essential transcription and translation processes can be accomplished during the extended aging period owe to the increased genomic content. The slow-cycle nature of polyploid cells allows them to avoid the lethal effects of chemoradiotherapy, which usually acts on cells in the mitotic state (Ogden et al., 2015). Zhang, S. et al. treated CoCl_2_-treated HEY control cells and PDCs with cisplatin (Zhang et al., 2014a). After cisplatin treatment, most of the small-sized control cells died, but PDCs enriched with CoCl_2_ survived (Zhang et al., 2014a). Furthermore, the number and size of PGCC in five cases of ovarian cancer after chemotherapy were significantly higher than those before chemotherapy (Zhang et al., 2014a). Radiation and chemotherapeutics can induce the formation of PDCs exhibiting high migratory, invasive, and proliferative abilities, by regulating the expression of EMT-related proteins (Fei et al., 2019a). When PDCs were treated with cisplatin for the second time, the recovery time (from cisplatin treatment to cells beginning proliferation) for the second treatment was shorter than that for the first treatment.

Neoadjuvant chemoradiation therapy (nCRT) is used to treat patients with locally advanced rectal cancer, who may experience a decline in tumor staging. Fei et al. collected 304 nCRT cases as well as 301 paired non-nCRT cases, and proved that more PGCCs, tumor budding, tumor emboli related to invasion, and metastasis appeared in the tumor tissue after nCRT. The average survival rate of patients who received nCRT was gradually worse than that of patients who did not. The rest interval was the optimal timing of surgery after nCRT. A rest interval of 60 days, tumor stage, recurrence and metastasis were significant predictors of survival in patients treated with nCRT, as assessed using multivariable analysis. PGCCs require a latency period to recover their viability and produce PDCs after chemoradiotherapy (Fei et al., 2019a). The optimal time for radical operation after nCRT should be within the latency period when PGCCs do not generate PDCs with high migration and invasion abilities. The choice of the operation time is very important for patients with locally advanced rectal cancer.

### Chemical reagents targeting PGCCs

Because of the critical role of PGCCs in the progression of malignant tumors, many therapeutic regimens targeting PGCCs have been used in clinical trials. PRL3 can induce a stem cell-like transcriptional program and promote cancer growth and survival (Zhou et al., 2017). Overexpression of PRL3 makes cells insensitive to DNA damage by inhibiting the ATM-mediated DNA damage, and helps them escape apoptosis under long-term genotoxic stress (Thura et al., 2021). PRL3-zumab blocks the role of PGCCs in tumors, by targeting the inhibition of PRL3, as an adjuvant immunotherapy. PRL3-zumab can remove circulating PRL3 + PGCCs, to prevent tumor recurrence and metastasis after tumor resection surgery. PGCCs can avoid the genotoxic effects of chemotherapy and radiotherapy, by entering a reversible static state (Demaria et al., 2017). Mitosis-independent PGCCs retreat from the therapy-induced senescence by means of depolyploidization, and generate PDCs for tumor propagation and disease recurrence (Liang et al., 2007; Al-Aidaroos and Zeng, 2010). PRL3-zumab targets “dormant” tumor cells, by inactivating classical chemo-resistant and residual PGCCs with self-renewal (Thura et al., 2021). Recently, PRL3-zumab was rapidly approved by the US Food and Drug Administration and the National Medical Products Administration for Phase II IND trials of solid PRL3 cancer in the United States of America.

PGCCs often contain excessive amounts of DNA and have an abnormal number of centrosomes. The combined application of centrosome disaggregation-related drugs and paclitaxel is believed to inhibit tumor resistance and recurrence (Ogden et al., 2015). Several existing centrosome depolymerization drugs exhibit long-term non-toxicity and strong anticancer activity (Coward and Harding, 2014), by separating two centrosome clusters formed by mitotic cancer cells at opposite spindle poles, which can force the spindle into a persistent multipolar, survival-incompatible configuration (Ogden et al., 2015). However, some clinical trials have shown that targeting mitotic inhibitors, such as Aurora kinase inhibitors, is not as effective as expected (Manchado et al., 2012; Benada and Macurek, 2015). The combined inactivation of Aurora A, B, and C can induce cancer cells to switch to endoreplication and form PGCCs. Inhibition of Aurora A or B alone does not strongly induce endoreplication, but instead leads to cell growth arrest and decreased cell numbers. Aurora inhibitors can protect non-small cell lung cancer cells (NSCLCs) from the cytotoxicity of antimitotic cancer drugs, but cannot protect cancer cells from the damage caused by drugs that target interphase cells. NSCLCs that adopt endoreplication can re-enter the proliferative cell cycle when Aurora inhibitors are no longer used (Tagal and Roth, 2021).

Tumor burden drops after radiation and biochemical treatment, which indicates that resistant tumors initiate from CSCs that are intrinsically resistant or develop resistance under therapeutic selection pressure (Amend et al., 2018). This recurrent tumor growth from single or multiple cells is supported by phylogenetic analyses (Greaves and Maley, 2012; Haffner et al., 2013; Maley et al., 2017). These cells are termed as keystone species. Keystone species can greatly influence the development of tumors and tumor environments (Amend et al., 2019). Therefore, we can prevent the evolution of tumors using such cells, similar to highly evolved treatment-resistant polyploid cells. Adipose and cartilage differentiation of PGCCs may provide opportunities for solid tumor therapy (Meng et al., 2001; Zhang et al., 2020). Zhang, S. et al. reported that PGCCs could be induced to differentiate into adipose, cartilage, and bone *in vitro* and *in vivo*. PGCCs cultured in adipogenesis medium showed strong staining with Oil Red O and expressed fatty acid-binding protein 4 (Zhang et al., 2014a). Adipocytes inside the xenografts derived from PGCCs undergoing adipogenic differentiation were positive for human-specific anti-vimentin immunohistochemical staining. When the animal abdominal cavity was injected with PGCCs cultured in chondrogenic medium, translucent tumor nodules similar to cartilage appeared within the tumor tissue, as confirmed by means of Safranin O/Fast Green histochemical staining and anti-eGFP, human-specific anti-osteopontin immunohistochemical staining.

### Tumor budding, micropapillary structure, and PDCs

“Tumor budding” refers to a cluster of tumor cells located at the invasion front (Hase et al., 1993; Ha et al., 2005)*,* which is composed of less than five cancer cells (Satoh et al., 2014; Manjula et al., 2015). There are more tumor buddings in poorly differentiated carcinomas than in well and moderately differentiated carcinomas (Ueno et al., 2004; Fukumoto et al., 2016). The micropapillary structure of adenocarcinoma is composed of a few of tumor cells. There are not vessels in the middle of micropapillary (Vardar et al., 2015). Micropapillary carcinoma can be observed in many types of malignant tumors, including colorectal cancer, lung cancer, bladder carcinoma, ovarian cancer, pancreatic cancer, and stomach cancer (Badyal et al., 2016; Bertz et al., 2016; Cao et al., 2016; Yang et al., 2016), and are associated with lymph and vascular vessel invasion and metastases.

Tumor budding and micropapillary structure are closely related to PGCCs and PDCs. Tumor budding refers to a single PGCC or several PDCs produced by PGCCs. PGCCs with PDCs can also form micropapillary structure. Both tumor budding and micropapillary structure can derive from PGCCs. PGCCs can be observed in most micropapillary structures. Zhang, S. et al. reported that single breast cancer cell line MCF-7 PGCCs derived from paclitaxel treatment could form papilla-like structures *in vitro* (Zhang et al., 2014d).

## Conclusion

**FIGURE 3:**
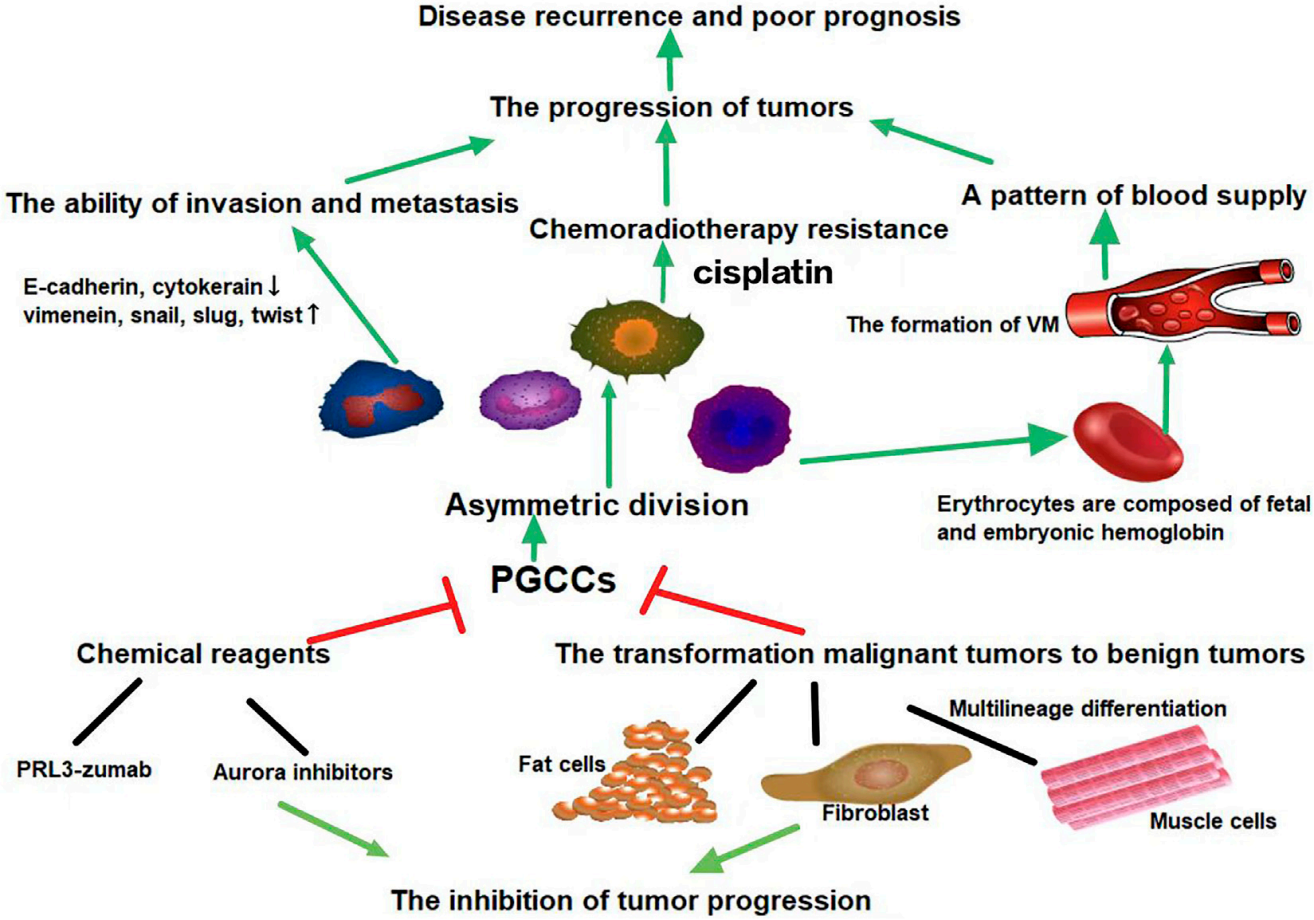
PGCCs can generate PDCs *via* asymmetric cell division. Daughter cells that originate from PGCCs can acquire strong abilities of invasion, metastasis, and chemoradiotherapy resistance. PGCCs can generate erythrocytes to form vasculogenic mimicry. The high oxygen affinity of fetal hemoglobin and embryonic hemoglobin derived from PGCCs allows tumor cells to survive in severe hypoxic conditions. Targeting relevant proteins or signaling pathways involved in the formation and transdifferentiation of adipose and cartilage of PGCCs may provide opportunities for solid tumor therapy. Green arrows represent potentiation, while red lines represent inhibitive effects.

PGCCs were previously thought to be senescent and non-proliferative. However, recent studies on PGCCs have revealed many of their poorly understood properties and functions. PGCCs can be induced by various stresses, including hypoxia, radiation, and chemotherapy. As a CSC population, PGCCs express CSC markers and produce PDCs. In addition, PGCCs with PDCs possess EMT phenotypes, which can enhance the invasion and metastasis of tumor cells. PGCCs can acquire therapeutic resistance and are responsible for tumor recurrence. Targeting PGCCs with CSC-like properties may provide opportunities for the treatment of malignant solid tumors (Figure 3).

## Abbreviations

ACD, asymmetric cell division; ATO, arsenic trioxide; CDK, cyclin-dependent kinase; Cdc42, cell cycle-related proteins and cell division control protein 42; CHK2, checkpoint Kinase 2; CoCl_2_, cobalt chloride; CSC, cancer stem cell; EMT, epithelial mesenchymal transition; GCM1, glial cell missing 1; JNK, c-Jun N-terminal kinase; LARC, locally advanced rectal cancer; NSCLC, non-small cell lung cancer; MAPK, mitogen-activated protein kinase; nCRT, neoadjuvant chemoradiation therapy; PGCCs, polyploid giant cancer cells; PDCs, PGCCs with daughter cells; PRL3, phosphatase of regenerating liver 3; VM, vasculogenic mimicry.

## References

[B1] Al-Aidaroos A. Zeng Q. (2010). PRL-3 phosphatase and cancer metastasis. J. Cell. Biochem. 111, 1087–1098. 10.1002/jcb.22913 21053359

[B2] Albitar M. Cash F. Peschle C. Liebhaber S. (1992). Developmental switch in the relative expression of the alpha 1- and alpha 2-globin genes in humans and in transgenic mice. Blood 79, 2471–2474. 10.1182/blood.v79.9.2471.2471 1571559

[B3] Amend S. de Groot A. Torga G. Axelrod H. Reyes D. Valkenburg K. (2018). Ten unanswered questions in cancer: "If this is true, what does it imply. Am. J. Clin. Exp. Urol. 6, 26–31. 29666828 PMC5902718

[B4] Amend S. R. Torga G. Lin K. C. Kostecka L. G. de Marzo A. Austin R. H. (2019). Polyploid giant cancer cells: Unrecognized actuators of tumorigenesis, metastasis, and resistance. Prostate 79, 1489–1497. 10.1002/pros.23877 31376205 PMC6706309

[B5] Baczyk D. Drewlo S. Proctor L. Dunk C. Lye S. Kingdom J. (2009). Glial cell missing-1 transcription factor is required for the differentiation of the human trophoblast. Cell. Death Differ. 16, 719–727. 10.1038/cdd.2009.1 19219068

[B6] Badyal R. K. Bal A. Das A. Singh G. (2016). Invasive micropapillary carcinoma of the breast: Immunophenotypic analysis and role of cell adhesion molecules (CD44 and E-cadherin) in nodal metastasis. Appl. Immunohistochem. Mol. Morphol. 24, 151–158. 10.1097/PAI.0000000000000167 26200840

[B7] Barr A. Cooper S. Heldt F. Butera F. Stoy H. Mansfeld J. (2017). DNA damage during S-phase mediates the proliferation-quiescence decision in the subsequent G1 via p21 expression. Nat. Commun. 8, 14728. 10.1038/ncomms14728 28317845 PMC5364389

[B8] Baugh E. Ke H. Levine A. Bonneau R. Chan C. (2018). Why are there hotspot mutations in the TP53 gene in human cancers? Cell. Death Differ. 25, 154–160. 10.1038/cdd.2017.180 29099487 PMC5729536

[B9] Ben-David U. Amon A. (2020). Context is everything: Aneuploidy in cancer. Nat. Rev. Genet. 21, 44–62. 10.1038/s41576-019-0171-x 31548659

[B10] Benada J. Macurek L. (2015). Targeting the checkpoint to kill cancer cells. Biomolecules 5, 1912–1937. 10.3390/biom5031912 26295265 PMC4598780

[B11] Bertz S. Wach S. Taubert H. Merten R. Krause F. S. Schick S. (2016). Micropapillary morphology is an indicator of poor prognosis in patients with urothelial carcinoma treated with transurethral resection and radiochemotherapy. Virchows Arch. 469, 339–344. 10.1007/s00428-016-1986-x 27392930

[B12] Bharadwaj R. Yu H. (2004). The spindle checkpoint, aneuploidy, and cancer. Oncogene 23, 2016–2027. 10.1038/sj.onc.1207374 15021889

[B13] Bourougaa K. Naski N. Boularan C. Mlynarczyk C. Candeias M. Marullo S. (2010). Endoplasmic reticulum stress induces G2 cell-cycle arrest via mRNA translation of the p53 isoform p53/47. Mol. Cell. 38, 78–88. 10.1016/j.molcel.2010.01.041 20385091

[B14] Boutros R. Lobjois V. Ducommun B. (2007). CDC25 phosphatases in cancer cells: Key players? Good targets? Nat. Rev. Cancer 7, 495–507. 10.1038/nrc2169 17568790

[B15] Bresnick A. Weber D. Zimmer D. (2015). S100 proteins in cancer. Nat. Rev. Cancer 15, 96–109. 10.1038/nrc3893 25614008 PMC4369764

[B16] Cao Y. Zhu L. Z. Jiang M. J. Yuan Y. (2016). Clinical impacts of a micropapillary pattern in lung adenocarcinoma: A review. Onco. Targets. Ther. 9, 149–158. 10.2147/OTT.S94747 26770064 PMC4706128

[B17] Chen J. Niu N. Zhang J. Qi L. Shen W. Donkena K. (2019). Polyploid giant cancer cells (PGCCs): The evil roots of cancer. Curr. Cancer Drug Targets 19, 360–367. 10.2174/1568009618666180703154233 29968537

[B18] Coward J. Harding A. (2014). Size does matter: Why polyploid tumor cells are critical drug targets in the war on cancer. Front. Oncol. 4, 123. 10.3389/fonc.2014.00123 24904834 PMC4033620

[B19] Das P. Pillai S. Rakib M. Khanam J. Gopalan V. Lam A. (2020). Plasticity of cancer stem cell: Origin and role in disease progression and therapy resistance. Stem Cell. Rev. Rep. 16, 397–412. 10.1007/s12015-019-09942-y 31965409

[B20] Demaria M. O'Leary M. Chang J. Shao L. Liu S. Alimirah F. (2017). Cellular senescence promotes adverse effects of chemotherapy and cancer relapse. Cancer Discov. 7, 165–176. 10.1158/2159-8290.CD-16-0241 27979832 PMC5296251

[B21] Dianat-Moghadam H. Heidarifard M. Jahanban-Esfahlan R. Panahi Y. Hamishehkar H. Pouremamali F. (2018). Cancer stem cells-emanated therapy resistance: Implications for liposomal drug delivery systems. J. Control. Release 288, 62–83. 10.1016/j.jconrel.2018.08.043 30184466

[B22] Edgar B. Zielke N. Gutierrez C. (2014). Endocycles: A recurrent evolutionary innovation for post-mitotic cell growth. Nat. Rev. Mol. Cell. Biol. 15, 197–210. 10.1038/nrm3756 24556841

[B23] Engeland K. (2018). Cell cycle arrest through indirect transcriptional repression by p53: I have a DREAM. Cell. Death Differ. 25, 114–132. 10.1038/cdd.2017.172 29125603 PMC5729532

[B24] Fei F. Liu K. Li C. Du J. Wei Z. Li B. (2020). Molecular mechanisms by which S100A4 regulates the migration and invasion of PGCCs with their daughter cells in human colorectal cancer. Front. Oncol. 10, 182. 10.3389/fonc.2020.00182 32154176 PMC7047322

[B25] Fei F. Qu J. Liu K. Li C. Wang X. Li Y. (2019). The subcellular location of cyclin B1 and CDC25 associated with the formation of polyploid giant cancer cells and their clinicopathological significance. Lab. Invest. 99, 483–498. 10.1038/s41374-018-0157-x 30487595

[B26] Fei F. Qu J. Zhang M. Li Y. Zhang S. (2017). S100A4 in cancer progression and metastasis: A systematic review. Oncotarget 8, 73219–73239. 10.18632/oncotarget.18016 29069865 PMC5641208

[B27] Fei F. Zhang D. Yang Z. Wang S. Wang X. Wu Z. (2015). The number of polyploid giant cancer cells and epithelial-mesenchymal transition-related proteins are associated with invasion and metastasis in human breast cancer. J. Exp. Clin. Cancer Res. 34, 158. 10.1186/s13046-015-0277-8 26702618 PMC4690326

[B28] Fei F. Zhang M. Li B. Zhao L. Wang H. Liu L. (2019). formation of polyploid giant cancer cells involves in the prognostic value of neoadjuvant chemoradiation in locally advanced rectal cancer. J. Oncol. 2019, 2316436. 10.1155/2019/2316436 31558902 PMC6735173

[B29] Fukumoto K. Kikuchi E. Mikami S. Ogihara K. Matsumoto K. Miyajima A. (2016). Tumor budding, a novel prognostic indicator for predicting stage progression in T1 bladder cancers. Cancer Sci. 107, 1338–1344. 10.1111/cas.12990 27317460 PMC5021027

[B30] Ganem N. Pellman D. (2007). Limiting the proliferation of polyploid cells. Cell. 131, 437–440. 10.1016/j.cell.2007.10.024 17981108

[B31] Gao C. Su Y. Koeman J. Haak E. Dykema K. Essenberg C. (2016). Chromosome instability drives phenotypic switching to metastasis. Proc. Natl. Acad. Sci. U. S. A. 113, 14793–14798. 10.1073/pnas.1618215113 27930335 PMC5187712

[B32] Geigl J. Obenauf A. Schwarzbraun T. Speicher M. (2008). Defining 'chromosomal instability. Trends Genet. 24, 64–69. 10.1016/j.tig.2007.11.006 18192061

[B33] Gheghiani L. Loew D. Lombard B. Mansfeld J. Gavet O. (2017). PLK1 activation in late G2 sets up commitment to mitosis. Cell. Rep. 19, 2060–2073. 10.1016/j.celrep.2017.05.031 28591578

[B34] Greaves M. Maley C. (2012). Clonal evolution in cancer. Nature 481, 306–313. 10.1038/nature10762 22258609 PMC3367003

[B35] Ha S. S. Choi H. J. Park K. J. Kim J. M. Kim S. H. Roh Y. H. (2005). Intensity of tumor budding as an index for the malignant potential in invasive rectal carcinoma. Cancer Res. Treat. 37, 177–182. 10.4143/crt.2005.37.3.177 19956500 PMC2785407

[B36] Haffner M. Mosbruger T. Esopi D. Fedor H. Heaphy C. Walker D. (2013). Tracking the clonal origin of lethal prostate cancer. J. Clin. Invest. 123, 4918–4922. 10.1172/JCI70354 24135135 PMC3809798

[B37] Harashima H. Dissmeyer N. Schnittger A. (2013). Cell cycle control across the eukaryotic kingdom. Trends Cell. Biol. 23, 345–356. 10.1016/j.tcb.2013.03.002 23566594

[B38] Hase K. Shatney C. Johnson D. Trollope M. Vierra M. (1993). Prognostic value of tumor "budding" in patients with colorectal cancer. Dis. Colon Rectum 36, 627–635. 10.1007/BF02238588 8348847

[B39] Haugwitz U. Wasner M. Wiedmann M. Spiesbach K. Rother K. Mössner J. (2002). A single cell cycle genes homology region (CHR) controls cell cycle-dependent transcription of the cdc25C phosphatase gene and is able to cooperate with E2F or Sp1/3 sites. Nucleic Acids Res. 30, 1967–1976. 10.1093/nar/30.9.1967 11972334 PMC113852

[B40] Hawkins N. Garriga G. (1998). Asymmetric cell division: from A to Z. Genes Dev. 12, 3625–3638. 10.1101/gad.12.23.3625 9851969

[B41] He K. Deora A. Xiong H. Ling Q. Weksler B. Niesvizky R. (2008). Endothelial cell annexin A2 regulates polyubiquitination and degradation of its binding partner S100A10/p11. J. Biol. Chem. 283, 19192–19200. 10.1074/jbc.M800100200 18434302 PMC2443646

[B42] He Z. Yu L. Luo S. Li M. Li J. Li Q. (2017). miR-296 inhibits the metastasis and epithelial-mesenchymal transition of colorectal cancer by targeting S100A4. BMC cancer 17, 140. 10.1186/s12885-017-3121-z 28209128 PMC5311719

[B43] Hitomi M. Stacey D. (2001). Ras-dependent cell cycle commitment during G2 phase. FEBS Lett. 490, 123–131. 10.1016/s0014-5793(01)02115-9 11223027

[B44] Ho V. Bunn H. (1996). Effects of transition metals on the expression of the erythropoietin gene: Further evidence that the oxygen sensor is a heme protein. Biochem. Biophys. Res. Commun. 223, 175–180. 10.1006/bbrc.1996.0865 8660366

[B45] Holland A. Cleveland D. (2009). Boveri revisited: Chromosomal instability, aneuploidy and tumorigenesis. Nat. Rev. Mol. Cell. Biol. 10, 478–487. 10.1038/nrm2718 19546858 PMC3154738

[B46] Horvitz H. Herskowitz I. (1992). Mechanisms of asymmetric cell division: Two bs or not two bs, that is the question. Cell. 68, 237–255. 10.1016/0092-8674(92)90468-r 1733500

[B47] Huehns E. Faroqui A. (1975). Oxygen dissociation properties of human embryonic red cells. Nature 254, 335–337. 10.1038/254335a0 1118015

[B48] Imai Y. Morishita S. Ikeda Y. Toyoda M. Ashizawa T. Yamamoto K. (1999). Immunohistochemical and molecular analysis of giant cell carcinoma of the pancreas: A report of three cases. Pancreas 18, 308–315. 10.1097/00006676-199904000-00013 10206490

[B49] Ishay-Ronen D. Diepenbruck M. Kalathur R. K. R. Sugiyama N. Tiede S. Ivanek R. (2019). Gain fat-lose metastasis: Converting invasive breast cancer cells into adipocytes inhibits cancer metastasis. Cancer Cell. 35, 17–32. 10.1016/j.ccell.2018.12.002 30645973

[B50] Jia L. Zhang S. Ye Y. Li X. Mercado-Uribe I. Bast R. C. Jr. (2012). Paclitaxel inhibits ovarian tumor growth by inducing epithelial cancer cells to benign fibroblast-like cells. Cancer Lett. 326, 176–182. 10.1016/j.canlet.2012.08.004 22902993 PMC3495569

[B51] Kazazian H. Woodhead A. (1973). Hemoglobin A synthesis in the developing fetus. N. Engl. J. Med. 289, 58–62. 10.1056/NEJM197307122890202 4710406

[B52] Keith B. Simon M. (2007). Hypoxia-inducible factors, stem cells, and cancer. Cell. 129, 465–472. 10.1016/j.cell.2007.04.019 17482542 PMC3150586

[B53] Kim E. Lee Y. Bae S. Lee J. Kim J. Lee Y. (2009). Heat shock factor 1-mediated aneuploidy requires a defective function of p53. Cancer Res. 69, 9404–9412. 10.1158/0008-5472.CAN-09-1411 19934326

[B54] Knerr I. Schubert S. Wich C. Amann K. Aigner T. Vogler T. (2005). Stimulation of GCMa and syncytin via cAMP mediated PKA signaling in human trophoblastic cells under normoxic and hypoxic conditions. FEBS Lett. 579, 3991–3998. 10.1016/j.febslet.2005.06.029 16004993

[B55] Lee H. Davidson J. Duronio R. (2009). Endoreplication: Polyploidy with purpose. Genes Dev. 23, 2461–2477. 10.1101/gad.1829209 19884253 PMC2779750

[B56] Li Z. Zheng M. Zhang H. Yang X. Fan L. Fu F. (2021). Arsenic trioxide promotes tumor progression by inducing the formation of PGCCs and embryonic hemoglobin in colon cancer cells. Front. Oncol. 11, 720814. 10.3389/fonc.2021.720814 34676163 PMC8523995

[B57] Liang C. Wang L. Chen C. Chen L. Chen Y. Chen H. (2010). GCM1 regulation of the expression of syncytin 2 and its cognate receptor MFSD2A in human placenta. Biol. Reprod. 83, 387–395. 10.1095/biolreprod.110.083915 20484742

[B58] Liang F. Liang J. Wang W. Sun J. Udho E. Zhang Z. (2007). PRL3 promotes cell invasion and proliferation by down-regulation of Csk leading to Src activation. J. Biol. Chem. 282, 5413–5419. 10.1074/jbc.M608940200 17192274

[B59] Lin C. Lin M. Chen H. (2005). Biochemical characterization of the human placental transcription factor GCMa/1. Biochem. Cell. Biol. = Biochimie Biol. Cell. 83, 188–195. 10.1139/o05-026 15864327

[B60] Liu G. Wang Y. Fei F. Wang X. Li C. Liu K. (2018). Clinical characteristics and preliminary morphological observation of 47 cases of primary anorectal malignant melanomas. Melanoma Res. 28, 592–599. 10.1097/CMR.0000000000000491 30080746

[B61] Liu J. (2018). The dualistic origin of human tumors. Semin. Cancer Biol. 53, 1–16. 10.1016/j.semcancer.2018.07.004 30040989 PMC6553492

[B62] Liu K. Zheng M. Lu R. Du J. Zhao Q. Li Z. (2020). The role of CDC25C in cell cycle regulation and clinical cancer therapy: A systematic review. Cancer Cell. Int. 20, 213. 10.1186/s12935-020-01304-w 32518522 PMC7268735

[B63] Liu K. Zheng M. Zhao Q. Zhang K. Li Z. Fu F. (2020). Different p53 genotypes regulating different phosphorylation sites and subcellular location of CDC25C associated with the formation of polyploid giant cancer cells. J. Exp. Clin. Cancer Res. 39, 83. 10.1186/s13046-020-01588-w 32393310 PMC7212590

[B64] Lopez-Beltran A. Eble J. Bostwick D. (2005). Pleomorphic giant cell carcinoma of the prostate. Arch. Pathol. Lab. Med. 129, 683–685. 10.1043/1543-2165(2005)129<0683:PGCCOT>2.0.CO;2 15859643

[B65] Lopez-Sanchez L. M. Jimenez C. Valverde A. Hernandez V. Penarando J. Martinez A. (2014). CoCl2, a mimic of hypoxia, induces formation of polyploid giant cells with stem characteristics in colon cancer. PLoS One 9, e99143. 10.1371/journal.pone.0099143 24932611 PMC4059626

[B66] Lorentzen M. (1980). Giant cell tumor of the ovary. Virchows Arch. A Pathol. Anat. Histol. 388, 113–122. 10.1007/BF00430681 7467120

[B67] Lu X. Kang Y. (2009). Cell fusion as a hidden force in tumor progression. Cancer Res. 69, 8536–8539. 10.1158/0008-5472.CAN-09-2159 19887616 PMC2783941

[B68] Lv H. Shi Y. Zhang L. Zhang D. Liu G. Yang Z. (2014). Polyploid giant cancer cells with budding and the expression of cyclin E, S-phase kinase-associated protein 2, stathmin associated with the grading and metastasis in serous ovarian tumor. BMC cancer 14, 576. 10.1186/1471-2407-14-576 25106448 PMC4137091

[B69] Maley C. Aktipis A. Graham T. Sottoriva A. Boddy A. Janiszewska M. (2017). Classifying the evolutionary and ecological features of neoplasms. Nat. Rev. Cancer 17, 605–619. 10.1038/nrc.2017.69 28912577 PMC5811185

[B70] Manchado E. Guillamot M. Malumbres M. (2012). Killing cells by targeting mitosis. Cell. Death Differ. 19, 369–377. 10.1038/cdd.2011.197 22223105 PMC3278741

[B71] Mani S. Guo W. Liao M. Eaton E. Ayyanan A. Zhou A. (2008). The epithelial-mesenchymal transition generates cells with properties of stem cells. Cell. 133, 704–715. 10.1016/j.cell.2008.03.027 18485877 PMC2728032

[B72] Manjula B. V. Augustine S. Selvam S. Mohan A. M. (2015). Prognostic and predictive factors in gingivo buccal complex squamous cell carcinoma: Role of tumor budding and pattern of invasion. Indian J. Otolaryngol. Head. Neck Surg. 67, 98–104. 10.1007/s12070-014-0787-2 25621262 PMC4298608

[B73] Margolis S. Perry J. Weitzel D. Freel C. Yoshida M. Haystead T. (2006). A role for PP1 in the Cdc2/Cyclin B-mediated positive feedback activation of Cdc25. Mol. Biol. Cell. 17, 1779–1789. 10.1091/mbc.e05-08-0751 16467385 PMC1415323

[B74] Matsuura K. Jigami T. Taniue K. Morishita Y. Adachi S. Senda T. (2011). Identification of a link between Wnt/β-catenin signalling and the cell fusion pathway. Nat. Commun. 2, 548. 10.1038/ncomms1551 22109522

[B75] Meng L. Zhou J. Sasano H. Suzuki T. Zeitoun K. M. Bulun S. E. (2001). Tumor necrosis factor alpha and interleukin 11 secreted by malignant breast epithelial cells inhibit adipocyte differentiation by selectively down-regulating CCAAT/enhancer binding protein alpha and peroxisome proliferator-activated receptor gamma: Mechanism of desmoplastic reaction. Cancer Res. 61, 2250–2255. 11280794

[B76] Moein S. Adibi R. da Silva Meirelles L. Nardi N. Gheisari Y. (2020). Cancer regeneration: Polyploid cells are the key drivers of tumor progression. Biochim. Biophys. Acta. Rev. Cancer 1874, 188408. 10.1016/j.bbcan.2020.188408 32827584

[B77] Molberg K. Heffess C. Delgado R. Albores-Saavedra J. (1998). Undifferentiated carcinoma with osteoclast-like giant cells of the pancreas and periampullary region. Cancer 82, 1279–1287. 10.1002/(sici)1097-0142(19980401)82:7<1279:aid-cncr10>3.0.co;2-3 9529019

[B78] Mosnier J. Balique J. (2000). Pleomorphic giant cell carcinoma of the esophagus with coexpression of cytokeratin and vimentin and neuroendocrine differentiation. Arch. Pathol. Lab. Med. 124, 135–138. 10.1043/0003-9985(2000)124<0135:PGCCOT>2.0.CO;2 10629146

[B79] Müllers E. Silva Cascales H. Burdova K. Macurek L. Lindqvist A. (2017). Residual Cdk1/2 activity after DNA damage promotes senescence. Aging Cell. 16, 575–584. 10.1111/acel.12588 28345297 PMC5418196

[B80] Müllers E. Silva Cascales H. Jaiswal H. Saurin A. Lindqvist A. (2014). Nuclear translocation of Cyclin B1 marks the restriction point for terminal cell cycle exit in G2 phase. Cell. cycleGeorget. Tex.) 13, 2733–2743. 10.4161/15384101.2015.945831 PMC4615111 25486360

[B81] Nai G. Amico E. Gimenez V. Guilmar M. (2005). Pancreatology : official journal of the International Association of Pancreatology, 5, 279–284.Osteoclast-like giant cell tumor of the pancreas associated with mucus-secreting adenocarcinoma. Case report and discussion of the histogenesis (IAP) . . Et. al. 10.1159/000085283 15849490

[B82] Nehme Z. Pasquereau S. Haidar Ahmad S. Coaquette A. Molimard C. Monnien F. (2021). Polyploid giant cancer cells, stemness and epithelial-mesenchymal plasticity elicited by human cytomegalovirus. Oncogene 40, 3030–3046. 10.1038/s41388-021-01715-7 33767437

[B83] Nehme Z. Pasquereau S. Haidar Ahmad S. El Baba R. Herbein G. (2022). Polyploid giant cancer cells, EZH2 and Myc upregulation in mammary epithelial cells infected with high-risk human cytomegalovirus. EBioMedicine 80, 104056. 10.1016/j.ebiom.2022.104056 35596973 PMC9121245

[B84] Niu N. Mercado-Uribe I. Liu J. (2017). Dedifferentiation into blastomere-like cancer stem cells via formation of polyploid giant cancer cells. Oncogene 36, 4887–4900. 10.1038/onc.2017.72 28436947 PMC5582213

[B85] Niu N. Zhang J. Zhang N. Mercado-Uribe I. Tao F. Han Z. (2016). Linking genomic reorganization to tumor initiation via the giant cell cycle. Oncogenesis 5, e281. 10.1038/oncsis.2016.75 27991913 PMC5177773

[B86] Nosrati N. Kapoor N. Kumar V. (2015). DNA damage stress induces the expression of ribosomal protein S27a gene in a p53-dependent manner. Gene 559, 44–51. 10.1016/j.gene.2015.01.014 25592822

[B87] O'Connor R. Hollowell C. Laven B. Yang X. Steinberg G. Zagaja G. (2002). Recurrent giant cell carcinoma of the bladder. J. Urology 167, 1784. 10.1097/00005392-200204000-00049 11912413

[B88] Ogden A. Rida P. C. Knudsen B. S. Kucuk O. Aneja R. (2015). Docetaxel-induced polyploidization may underlie chemoresistance and disease relapse. Cancer Lett. 367, 89–92. 10.1016/j.canlet.2015.06.025 26185000 PMC4813805

[B89] Olenyuk B. Zhang G. Klco J. Nickols N. Kaelin W. Dervan P. (2004). Inhibition of vascular endothelial growth factor with a sequence-specific hypoxia response element antagonist. Proc. Natl. Acad. Sci. U. S. A. 101, 16768–16773. 10.1073/pnas.0407617101 15556999 PMC534742

[B90] Otto S. P. Whitton J. (2000). Polyploid incidence and evolution. Annu. Rev. Genet. 34, 401–437. 10.1146/annurev.genet.34.1.401 11092833

[B91] Øvrebø J. Edgar B. (2018). Polyploidy in tissue homeostasis and regeneration. Cambridge, England: Development, 145. 10.1242/dev.156034 PMC10682953 30021843

[B92] Peschle C. Mavilio F. Carè A. Migliaccio G. Migliaccio A. Salvo G. (1985). Haemoglobin switching in human embryos: Asynchrony of zeta-alpha and epsilon-gamma-globin switches in primitive and definite erythropoietic lineage. Nature 313, 235–238. 10.1038/313235a0 2578614

[B93] Pienta K. J. Hammarlund E. U. Axelrod R. Brown J. S. Amend S. R. (2020). Poly-aneuploid cancer cells promote evolvability, generating lethal cancer. Evol. Appl. 13, 1626–1634. 10.1111/eva.12929 32952609 PMC7484876

[B94] Pokieser W. Ulrich W. Neuhold N. Hoebling W. Hurtl I. (2003). Giant cells in poorly differentiated (insular) carcinoma of the thyroid. Acta Cytol. 47, 108–110. 12585043

[B95] Sakai Y. Kupelioglu A. Yanagisawa A. Yamaguchi K. Hidaka E. Matsuya S. (2000). Origin of giant cells in osteoclast-like giant cell tumors of the pancreas. Hum. Pathol. 31, 1223–1229. 10.1053/hupa.2000.18491 11070115

[B96] Satoh K. Nimura S. Aoki M. Hamasaki M. Koga K. Iwasaki H. (2014). Tumor budding in colorectal carcinoma assessed by cytokeratin immunostaining and budding areas: Possible involvement of c-met. Cancer Sci. 105, 1487–1495. 10.1111/cas.12530 25220207 PMC4462370

[B97] Seftor R. Seftor E. Kirschmann D. Hendrix M. (2002). Targeting the tumor microenvironment with chemically modified tetracyclines: Inhibition of laminin 5 gamma2 chain promigratory fragments and vasculogenic mimicry. Mol. Cancer Ther. 1, 1173–1179. 12479698

[B98] Seftor R. Seftor E. Koshikawa N. Meltzer P. Gardner L. Bilban M. (2001). Cooperative interactions of laminin 5 gamma2 chain, matrix metalloproteinase-2, and membrane type-1-matrix/metalloproteinase are required for mimicry of embryonic vasculogenesis by aggressive melanoma. Cancer Res. 61, 6322–6327. 11522618

[B99] Shams I. Avivi A. Nevo E. (2004). Hypoxic stress tolerance of the blind subterranean mole rat: Expression of erythropoietin and hypoxia-inducible factor 1 alpha. Proc. Natl. Acad. Sci. U. S. A. 101, 9698–9703. 10.1073/pnas.0403540101 15210955 PMC470738

[B100] Sheltzer J. Ko J. Replogle J. Habibe Burgos N. Chung E. Meehl C. (2017). Single-chromosome gains commonly function as tumor suppressors. Cancer Cell. 31, 240–255. 10.1016/j.ccell.2016.12.004 28089890 PMC5713901

[B101] Shen R. Wen P. (2004). Clear cell renal cell carcinoma with syncytial giant cells: A case report and review of the literature. Arch. Pathol. Lab. Med. 128, 1435–1438. 10.1043/1543-2165(2004)128<1435:CCRCCW>2.0.CO;2 15578891

[B102] Song Y. Zhao Y. Deng Z. Zhao R. Huang Q. (2021). Stress-induced polyploid giant cancer cells: Unique way of formation and non-negligible characteristics. Front. Oncol. 11, 724781. 10.3389/fonc.2021.724781 34527590 PMC8435787

[B103] St Clair S. Giono L. Varmeh-Ziaie S. Resnick-Silverman L. Liu W. Padi A. (2004). DNA damage-induced downregulation of Cdc25C is mediated by p53 via two independent mechanisms: One involves direct binding to the cdc25C promoter. Mol. Cell. 16, 725–736. 10.1016/j.molcel.2004.11.002 15574328

[B104] Stacey D. W. (2003). Cyclin D1 serves as a cell cycle regulatory switch in actively proliferating cells. Curr. Opin. Cell. Biol. 15, 158–163. 10.1016/s0955-0674(03)00008-5 12648671

[B105] Strick R. Ackermann S. Langbein M. Swiatek J. Schubert S. Hashemolhosseini S. (2007). Proliferation and cell-cell fusion of endometrial carcinoma are induced by the human endogenous retroviral Syncytin-1 and regulated by TGF-beta. J. Mol. Med. 85, 23–38. 10.1007/s00109-006-0104-y 17066266

[B106] Sur S. Agrawal D. (2016). Phosphatases and kinases regulating CDC25 activity in the cell cycle: Clinical implications of CDC25 overexpression and potential treatment strategies. Mol. Cell. Biochem. 416, 33–46. 10.1007/s11010-016-2693-2 27038604 PMC4862931

[B107] Tagal V. Roth M. G. (2021). Loss of Aurora kinase signaling allows lung cancer cells to adopt endoreplication and form polyploid giant cancer cells that resist antimitotic drugs. Cancer Res. 81, 400–413. 10.1158/0008-5472.CAN-20-1693 33172929 PMC8788927

[B108] Taylor A. Shih J. Ha G. Gao G. Zhang X. Berger A. (2018). Genomic and functional approaches to understanding cancer aneuploidy. Cancer Cell. 33, 676–689. e3. 10.1016/j.ccell.2018.03.007 29622463 PMC6028190

[B109] Thura M. Ye Z. Al-Aidaroos A. Q. Xiong Q. Ong J. Y. Gupta A. (2021). PRL3 induces polypoid giant cancer cells eliminated by PRL3-zumab to reduce tumor relapse. Commun. Biol. 4, 923. 10.1038/s42003-021-02449-8 34326464 PMC8322210

[B110] Toyoshima-Morimoto F. Taniguchi E. Nishida E. (2002). Plk1 promotes nuclear translocation of human Cdc25C during prophase. EMBO Rep. 3, 341–348. 10.1093/embo-reports/kvf069 11897663 PMC1084057

[B111] Ueno H. Price A. B. Wilkinson K. H. Jass J. R. Mochizuki H. Talbot I. C. (2004). A new prognostic staging system for rectal cancer. Ann. Surg. 240, 832–839. 10.1097/01.sla.0000143243.81014.f2 15492565 PMC1356489

[B112] Vardar E. Yardim B. G. Vardar R. Olmez M. (2015). Primary gastric invasive micropapillary carcinoma: A case report. Turk Patoloji Derg. 31, 219–222. 10.5146/tjpath.2014.01246 26456969

[B113] Vasudevan A. Baruah P. Smith J. Wang Z. Sayles N. Andrews P. (2020). Single-Chromosomal gains can function as metastasis suppressors and promoters in colon cancer. Dev. Cell. 52, 413–428. e6. 10.1016/j.devcel.2020.01.034 32097652 PMC7354079

[B114] Vasudevan A. Schukken K. M. Sausville E. L. Girish V. Adebambo O. A. Sheltzer J. M. (2021). Aneuploidy as a promoter and suppressor of malignant growth. Nat. Rev. Cancer 21, 89–103. 10.1038/s41568-020-00321-1 33432169

[B115] Wang X. Zheng M. Fei F. Li C. Du J. Liu K. (2019). EMT-related protein expression in polyploid giant cancer cells and their daughter cells with different passages after triptolide treatment. Med. Oncol. 36, 82. 10.1007/s12032-019-1303-z 31407170

[B116] Yang M. Wu M. Chiou S. Chen P. Chang S. Liu C. (2008). Direct regulation of TWIST by HIF-1alpha promotes metastasis. Nat. Cell. Biol. 10, 295–305. 10.1038/ncb1691 18297062

[B117] Yang Y. L. Liu B. B. Zhang X. Fu L. (2016). Invasive micropapillary carcinoma of the breast: An update. Arch. Pathol. Lab. Med. 140, 799–805. 10.5858/arpa.2016-0040-RA 27472238

[B118] Yang Z. Yao H. Fei F. Li Y. Qu J. Li C. (2018). Generation of erythroid cells from polyploid giant cancer cells: Re-thinking about tumor blood supply. J. Cancer Res. Clin. Oncol. 144, 617–627. 10.1007/s00432-018-2598-4 29417259 PMC11813446

[B119] Yu C. Shen K. Lin M. Chen P. Lin C. Chang G. (2002). GCMa regulates the syncytin-mediated trophoblastic fusion. J. Biol. Chem. 277, 50062–50068. 10.1074/jbc.M209316200 12397062

[B120] Zannini L. Delia D. Buscemi G. (2014). CHK2 kinase in the DNA damage response and beyond. J. Mol. Cell. Biol. 6, 442–457. 10.1093/jmcb/mju045 25404613 PMC4296918

[B121] Zhang D. Wang Y. Zhang S. (2014). Asymmetric cell division in polyploid giant cancer cells and low eukaryotic cells. Biomed. Res. Int. 2014, 432652. 10.1155/2014/432652 25045675 PMC4089188

[B122] Zhang H. Ma H. Yang X. Fan L. Tian S. Niu R. (2021). Cell fusion-related proteins and signaling pathways, and their roles in the development and progression of cancer. Front. Cell. Dev. Biol. 9, 809668. 10.3389/fcell.2021.809668 35178400 PMC8846309

[B123] Zhang J. Hou S. Gu J. Tian T. Yuan Q. Jia J. (2018). S100A4 promotes colon inflammation and colitis-associated colon tumorigenesis. Oncoimmunology 7, e1461301. 10.1080/2162402X.2018.1461301 30221056 PMC6136879

[B124] Zhang K. Yang X. Zhao Q. Li Z. Fu F. Zhang H. (2020). Molecular mechanism of stem cell differentiation into adipocytes and adipocyte differentiation of malignant tumor. Stem Cells Int. 2020, 8892300. 10.1155/2020/8892300 32849880 PMC7441422

[B125] Zhang L. Ding P. Lv H. Zhang D. Liu G. Yang Z. (2014). Number of polyploid giant cancer cells and expression of EZH2 are associated with VM formation and tumor grade in human ovarian tumor. Biomed. Res. Int. 2014, 903542. 10.1155/2014/903542 25025074 PMC4082869

[B126] Zhang L. Wu C. Hoffman R. (2015). Prostate cancer heterogeneous high-metastatic multi-organ-colonizing chemo-resistant variants selected by serial metastatic passage in nude mice are highly enriched for multinucleate giant cells. PloS one 10, e0140721. 10.1371/journal.pone.0140721 26536025 PMC4633180

[B127] Zhang S. Mercado-Uribe I. Hanash S. Liu J. (2013). iTRAQ-based proteomic analysis of polyploid giant cancer cells and budding progeny cells reveals several distinct pathways for ovarian cancer development. PLoS One 8, e80120. 10.1371/journal.pone.0080120 24348907 PMC3858113

[B128] Zhang S. Mercado-Uribe I. Liu J. (2013). Generation of erythroid cells from fibroblasts and cancer cells *in vitro* and *in vivo* . Cancer Lett. 333, 205–212. 10.1016/j.canlet.2013.01.037 23376638 PMC3760787

[B129] Zhang S. Mercado-Uribe I. Liu J. (2014). Tumor stroma and differentiated cancer cells can be originated directly from polyploid giant cancer cells induced by paclitaxel. Int. J. Cancer 134, 508–518. 10.1002/ijc.28319 23754740 PMC4175522

[B130] Zhang S. Mercado-Uribe I. Xing Z. Sun B. Kuang J. Liu J. (2014). Generation of cancer stem-like cells through the formation of polyploid giant cancer cells. Oncogene 33, 116–128. 10.1038/onc.2013.96 23524583 PMC3844126

[B131] Zhang S. Zhang D. Sun B. (2007). Vasculogenic mimicry: Current status and future prospects. Cancer Lett. 254, 157–164. 10.1016/j.canlet.2006.12.036 17306454

[B132] Zhang X. Hua R. Wang X. Huang M. Gan L. Wu Z. (2016). Identification of stem-like cells and clinical significance of candidate stem cell markers in gastric cancer. Oncotarget 7, 9815–9831. 10.18632/oncotarget.6890 26769843 PMC4891086

[B133] Zhao Q. Zhang K. Li Z. Zhang H. Fu F. Fu J. (2021). High migration and invasion ability of PGCCs and their daughter cells associated with the nuclear localization of S100A10 modified by SUMOylation. Front. Cell. Dev. Biol. 9, 696871. 10.3389/fcell.2021.696871 34336846 PMC8322665

[B134] Zhou J. Chan Z. L. Bi C. Lu X. Chong P. S. Chooi J. Y. (2017). LIN28B activation by PRL-3 promotes leukemogenesis and a stem cell-like transcriptional program in AML. Mol. Cancer Res. 15, 294–303. 10.1158/1541-7786.MCR-16-0275-T 28011885

[B135] Zielke N. Edgar B. DePamphilis M. (2013). Cold Spring Harb. Perspect. Biol. 5, a012948. 10.1101/cshperspect.a012948 23284048 PMC3579398

